# Application of cofactors in the regulation of microbial metabolism: A state of the art review

**DOI:** 10.3389/fmicb.2023.1145784

**Published:** 2023-04-11

**Authors:** Yang Sun, Ting Zhang, Bingqian Lu, Xiangfei Li, Ling Jiang

**Affiliations:** ^1^State Key Laboratory of Materials-Oriented Chemical Engineering, College of Food Science and Light Industry, Nanjing Tech University, Nanjing, China; ^2^Engineering Laboratory for Industrial Microbiology Molecular Beeding of Anhui Province, College of Biologic and Food Engineering, Anhui Polytechnic University, Wuhu, China

**Keywords:** cofactors, metabolic engineering, regulatory strategies, microbial cell factory, biological manufacturing

## Abstract

Cofactors are crucial chemicals that maintain cellular redox balance and drive the cell to do synthetic and catabolic reactions. They are involved in practically all enzymatic activities that occur in live cells. It has been a hot research topic in recent years to manage their concentrations and forms in microbial cells by using appropriate techniques to obtain more high-quality target products. In this review, we first summarize the physiological functions of common cofactors, and give a brief overview of common cofactors acetyl coenzyme A, NAD(P)H/NAD(P)^+^, and ATP/ADP; then we provide a detailed introduction of intracellular cofactor regeneration pathways, review the regulation of cofactor forms and concentrations by molecular biological means, and review the existing regulatory strategies of microbial cellular cofactors and their application progress, to maximize and rapidly direct the metabolic flux to target metabolites. Finally, we speculate on the future of cofactor engineering applications in cell factories.

**Figure:**
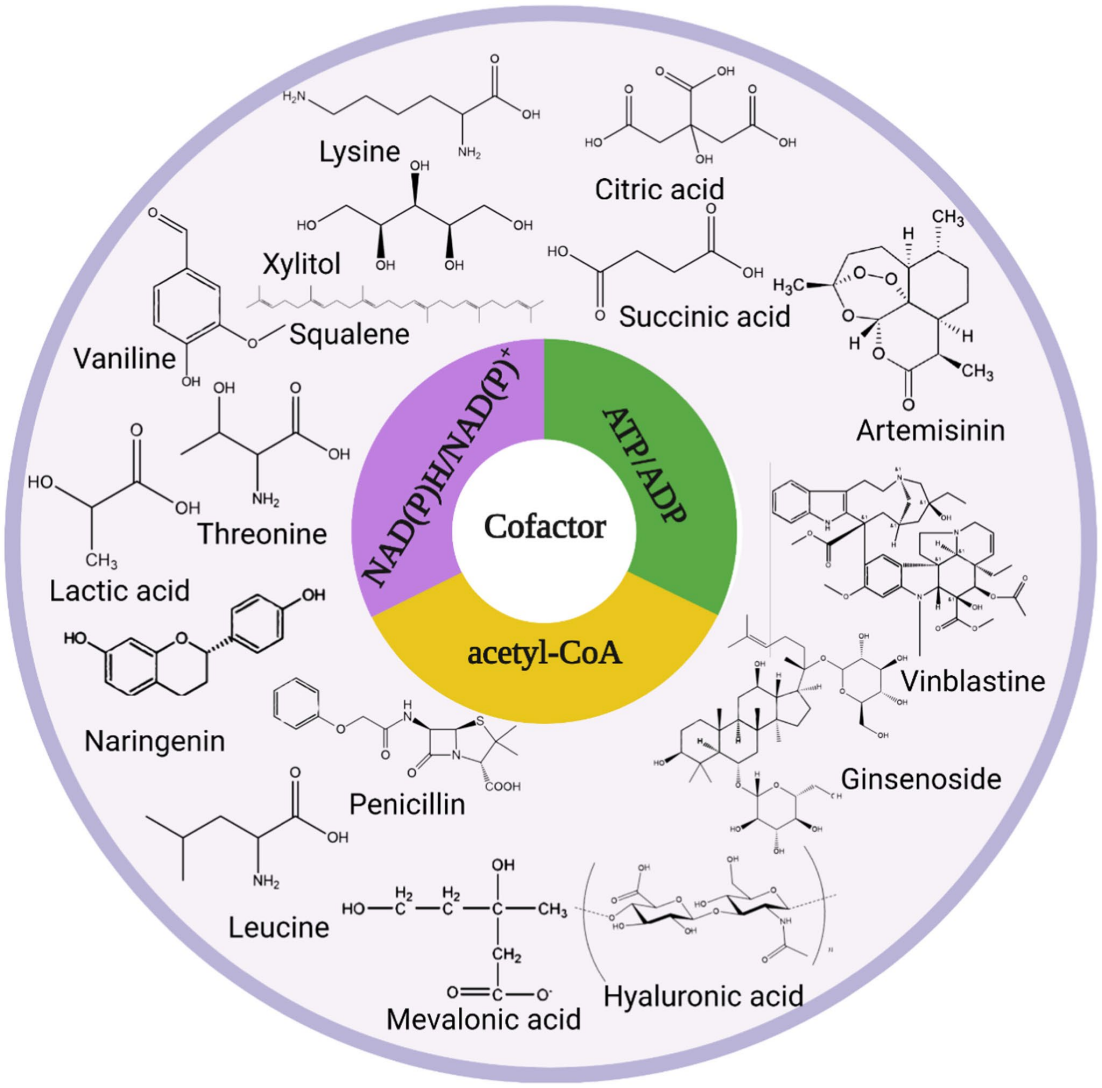
Graphical Abstract

Graphical Abstract

## Introduction

Traditional petrochemical manufacturing has several disadvantages, the most evident of which is irreversible environmental contamination, which is a significant breach of the original aim of harmonious cohabitation between humans and nature. Therefore, bioconversion, which allows the production of chemical products from renewable carbon sources, has become an ideal technology (Bruschi et al., 2011). With the booming development of synthetic biology and metabolic engineering technologies in recent years, the application of microbial cell factories to manufacture diverse high-value-added compounds has become a research hotspot (Zhang et al., 2018; Liu et al., 2021b). Artemisinin (Paddon et al., 2013), vinblastine (Zhang J. et al., 2022), ginsenoside (Wang P. et al., 2019), hyaluronic acid (Wang Y. et al., 2020) polyketides(Wang W. et al., 2020), and squalene (Zhu et al., 2021) have all been full-synthesized or semi-synthesized by researchers.

Cofactors are frequently required for cell-based biotransformation processes (Duetz et al., 2001; Drepper et al., 2006), proteases have a chemical structure that comprises polypeptides and certain non-protein structures, and one refers to these non-protein structures such as metal ions, metal–organic/inorganic complexes, and organic small molecules collectively as cofactors. Cofactors are required for enzymes to fulfill their catalytic functions, and they combine with the original enzyme to generate the entire enzyme, which then performs the enzyme’s normal physiological responsibilities (McNaught and Wilkinson, 1997). Common cofactors include acetyl coenzyme A (acetyl-CoA), NAD(P)H/NAD(P)^+^, and ATP/ADP, of NAD(P)H/NAD(P)^+^ and ATP/ADP-dependent metabolic reactions involving up to 1,610 enzymes such as transferases, oxidoreductases, lyases, ligases, isomerase, and hydrolases. Acetyl-CoA, NAD(P)H/NAD(P)^+^, and ATP/ADP play direct roles in the metabolism and transport of sugars, proteins, and lipids (Liu et al., 2018), which in turn affect cellular physiological functions. When these cofactors are created and consumed by cellular metabolism, their redox state is disrupted, resulting in consequences such as sluggish cell growth and decreased biosynthesis. Adjusting the concentration and form of cofactors can alter the distribution of material metabolic flux, push metabolism toward maximum target products, accelerate glycolysis, and preserve the complex intracellular structure (Selles et al., 2018). Modification strategies such as modification of endogenous or exogenous cofactor metabolic pathways, conversion between cofactors, and fine-tuning of transcription factors (Wang et al., 2017) can effectively maintain intracellular redox balance, and cofactor engineering has emerged as a powerful tool for increasing production capacity (Li et al., 2018). In this article, we summarize the physiological functions of common cofactors as well as the existing regulatory strategies of microbial cellular cofactors, as well as the progress of their applications, to serve as a reference for the efficient synthesis of target metabolites and to look ahead to the future development of cofactor engineering applications in cell factories.

## Cofactor physiological functions

**Figure 1:**
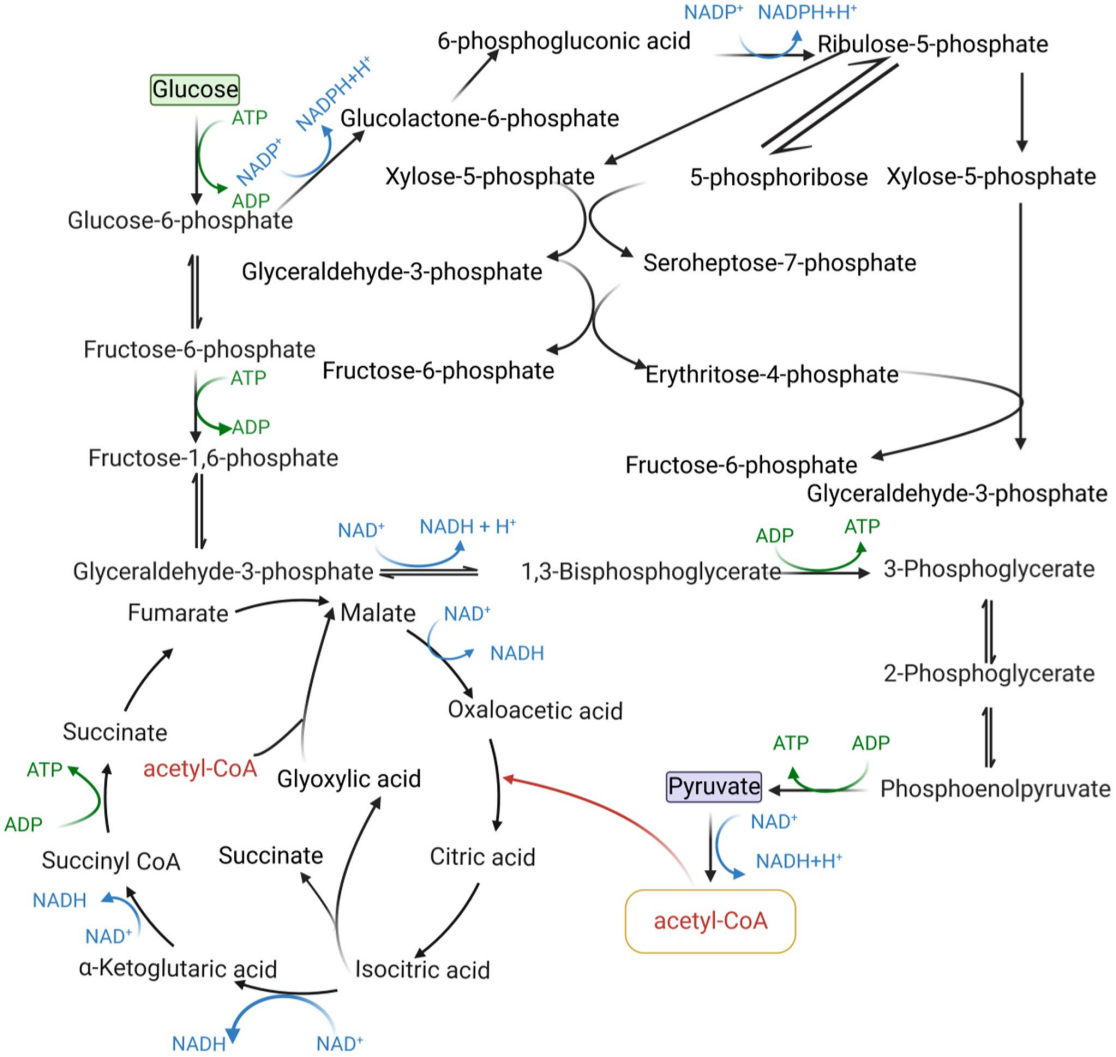
Pathway of cofactor production in microbial metabolism.

The intracellular redox state is closely related to cofactors, which can influence cellular metabolism, signal transduction, and material transport through cofactor regeneration, thereby affecting cellular physiological functions, and cofactors are also carriers of biological redox reactions and important factors in energy transfer (Wang et al., 2013). Cofactors can be divided into three broad categories based on their chemical structure and their role in enzyme-catalyzed reactions as described below: (i) catalytic cofactors, which are found in the active center of the enzyme and work together to catalyze the reaction. (ii) carrier cofactors, which are frequently used as carriers of electrons and atoms. (iii) substrate cofactors, which serve as raw materials for the synthesis of certain specific biological small molecular compounds and decrease as the reaction proceeds (Kulkarni and Deshpande, 2007). This article introduces three cofactors that play important roles in microbial cell metabolism: acetyl-CoA, NAD(P)H/NAD(P)^+^, and ATP/ADP, and their metabolic pathway in microbial cell metabolism is shown in Figure 1.

### Acetyl coenzyme A

The acetyl-CoA synthetic pathway connects the various metabolic reactions occurring in the cytoplasm, nucleus, and mitochondria and can provide the cell with both a carbon source and energy (Wang J. et al., 2020). Second, acetyl-CoA serves as a precursor for the synthesis of isoprenoids, fatty acids and their derivatives, terpenoids, flavonoids, polyketides, etc. For example, acetyl-CoA generates isoprene and terpenoids through the MVA pathway (Liu H. et al., 2020). Acetyl-CoA can modify post-translational proteins and regulate cellular protein biological activity and stability (Su et al., 2016). Acetyl-CoA maintains the balance between cell proliferation and apoptosis by acting as both a metabolic intermediate and a second messenger (Wagner et al., 2017).

### NAD(P)H/NAD(P)^+^

Intracellular NADH is mainly derived from glycolysis, fatty acid oxidation, and the tricarboxylic acid cycle, and pyruvate is converted to acetyl-CoA and NADH under aerobic conditions catalyzed by the pyruvate dehydrogenase complex. NAD(P)H/NAD(P)^+^ has a wide range of functions and can participate in approximately 1,500 enzymatic reactions in microbial metabolism, more than 100 of which have been linked to NADPH (Lee et al., 2013). NAD(P)H and NAD(P)^+^ play important roles in microbial cells as electron donors and acceptors, generating energy through electron transfer and participating in aerobic respiratory fermentation (Xu et al., 2020). Under aerobic conditions, NADH generates ATP *via* the electron transport chain (Curtabbi and Enriquez, 2022). Under anaerobic conditions, NADH is oxidized in the presence of acetaldehyde dehydrogenase and lactate dehydrogenase, when ATP is produced mainly through phosphorylation at the substrate level. NADPH homeostasis is regulated by a variety of signaling pathways and several metabolic enzymes that occur when cancer cells are altered and regulating NADPH in cancer cells is beneficial for cancer cell elimination (Pietrocola et al., 2015).

### ATP/ADP

Substrate-level phosphorylation is an important pathway for ATP production, especially when oxidative phosphorylation is restricted. Two moles of ATP are produced per mole of glucose in the glycolytic pathway catalyzed by glycerol-3-acid phosphokinase and pyruvate kinase. The cofactor ATP/ADP, which is generated by substrate-level and oxidative phosphorylation, can enter the metabolic network of microorganisms in a variety of forms such as substrates, products, activators, and inhibitors, control the physiological functions of the cell and contribute to the formation of the cytoskeletal system (Zhou et al., 2009). ATP can power almost all cells, and sufficient ATP must be created to allow normal cell biosynthesis and cell maintenance (Yu et al., 2018). *Aspergillus niger* (*A. niger*) strain DS03043 is capable of producing glucoamylase, and 62% of the total ATP produced in its cells is used for biomass formation, while the remaining 27% may be used for non-growth maintenance (Lu et al., 2015). ATP can regulate the rate of cellular metabolism. The rate of glycolysis is determined by the demand for total cellular ATP rather than the expression of glycolysis-related enzymes (Hackett et al., 2016). The activity of essential enzymes in the tricarboxylic acid cycle is inhibited when ATP concentration is too high (Wang W. et al., 2020). ATP is a metabolic activator of lactate dehydrogenase (Willquist and van Niel, 2010).

## Regulation of cofactor metabolic balance and its application

**Figure 2:**
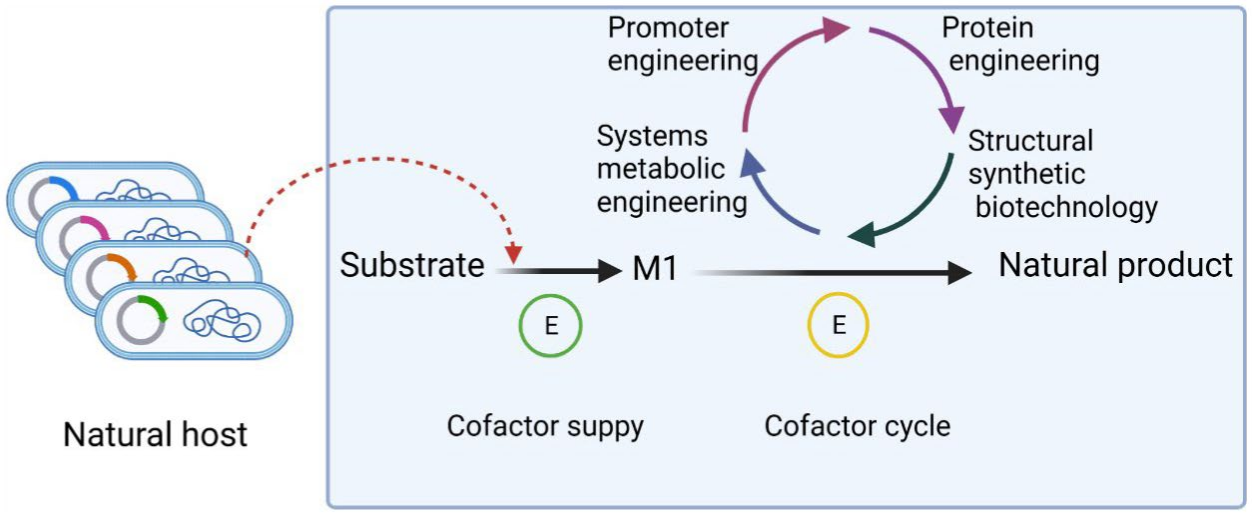
Process of product production in microbial metabolism. Cofactors play an important role in biosynthesis. First of all, the substrate was used to generate M1, which is necessary for the growth of microorganisms, through the combined action of cofactors, etc. The precursor M1 is then used as the precursor to synthesize substances with more complex chemical structure and no obvious physiological function for the microorganism. The cofactors of the whole synthesis process can be regulated by promoter engineering, protein engineering, structural synthetic biotechnology, system metabolic engineering and so on. These strategies make cofactor transition quickly and help to improve the cofactors balance.

Metabolic engineering is based on intracellular biochemical reactions that regulate the concentration and form of the cellular metabolic network to maximize the production of the target product. The flow of product generation in microbial metabolism is shown in Figure 2.

### Acetyl coenzyme A

Acetyl-CoA, as an intermediary product of cellular metabolism, serves as a critical hub in microbial metabolism and is connected to the glycolytic, TCA cycle, amino acid, and fatty acid synthesis pathways (Farmer and Liao, 1997; Sagmeister et al., 2014). The most common current regulatory strategy regarding acetyl-CoA is to regulate it through the acetate pathway. This section focuses on acetyl-CoA’s metabolic regulatory strategy and its application in biosynthesis. Some of the regulatory strategies of acetyl-CoA and their applications are listed in Table 1.

**Table 1:** Acetyl-CoA regulatory strategy.

Cofactors	Chassis cells	Metabolites	Production	Ref.
Acetyl-CoA	*Escherichia coli*	Malonyl Coenzyme A	More than 4-fold increase in malonyl coenzyme A synthesis yield compared to the control	Zha et al. (2009)
Acetyl-CoA	*Yarrowia lipolytica*	Lipids	Lipid content was then raised to 25.7% by overexpression of ACC and FAS	Chen et al. (2021)
Acetyl-CoA	*E. coli*	α-Ketoglutaric acid	Alpha-ketoglutaric acid yield up to 28.54 g/L	Chen et al. (2020)
Acetyl-CoA	*E. coli*	3-Hydroxypropionate	1.9-fold higher yield of 3-hydroxypropionate	Liu et al. (2017)
Acetyl-CoA	*Corynebacterium glutamicum*	5-aminolevulinic acid	A maximum yield of 5.6 g/L of 5-aminolevulinic acid.	Ge et al. (2021)
Acetyl-CoA	*Saccharomyces cerevisiae*	CoA nucleotides	The level of CoA nucleotides was 15-fold increased compared to control	Olzhausen et al. (2021)
Acetyl-CoA	*S. cerevisiae*	Mevalonate	Increase production to 3,830 ± 120 mg/L	Wegner et al. (2021)
Acetyl-CoA	*E. coli*	Mevalonate	Production of 8.0 g/L mevalonate from 20 g/L glucose	Satowa et al. (2020)
Acetyl-CoA	*S. cerevisiae*	Nerolidol	Nerolidol production was improved 2-fold to in flask cultivation	Hayat et al. (2021)
Acetyl-CoA	*E. coli*	Naringenin	Naringin production increased 27.2 fold	Kim et al. (2022)
Acetyl-CoA	*S. cerevisiae*	Lycopene	68 mg/g CDW lycopene	Su et al. (2022)

#### Regulation of acetic acid metabolic pathway

Acetyl-CoA and acetic acid can be converted to each other, acetic acid is catalyzed by acetyl-CoA synthase (ACS) to generate acetyl-CoA, while acetyl-CoA is catalyzed by phosphoryl acetyltransferase and acetic acid kinase to produce acetic acid. By overexpressing ACS in *E. coli*, Zha et al. (2009) not only reduced acetic acid accumulation but also enhanced the metabolic flux of acetyl-CoA to the target product. Chen X. et al. (2020) overexpressed ACS to improve the availability of acetyl-CoA, resulting in α-keto glutaric acid production of up to 28.54 g/L. Chen et al. (2021) accumulated 9.2% of microbial lipids from acetate in shake flask fermentation by overexpressing ACS in *Yarrowia lipolytica* (*Y. lipolytica*). The lipid content was then raised to 25.7% by overexpression of acetyl-CoA carboxylase (ACC) and fatty acid synthase (FAS) in *Y. lipolytica*.

#### Regulation of glyoxalate metabolic pathway

Isocitrate lyase, isocitrate dehydrogenase, and malate synthase are key enzymes in the glyoxylate cycle. IclR can inhibit the expression of the *aceBAK* operon, which encodes these three enzymes. The *iclR* gene was knocked out, which activated the glyoxylate branch and enhanced the acetyl-CoA synthesis metabolic flux, resulting in a 1.9-fold increase in the yield of 3-hydroxypropionate with acetyl-CoA as a precursor (Liu et al., 2017). Ge et al. (2021) obtained 3.86 g/L of 5-aminolevulinic acid in *Corynebacterium glutamicum* (*C. glutamicum*) by alleviating the competition between glyoxylate and TCA cycle and knocking out the gene encoding isocitrate dehydrogenase, with a maximum yield of 5.6 g/L of 5-aminolevulinic acid after optimization of fermentation conditions.

#### Other metabolic regulatory pathways

Acetyl-CoA, as an important hub of biosynthesis, can be regulated in a variety of ways such as overexpression of related pathway genes, modification of related pathway enzymes, the introduction of exogenous metabolic pathways, and redistribution of metabolic fluxes in order to obtain more target products.

Zhang M. et al. (2022) expressed the phosphoketolase encoding gene (*pK*) and the pyruvate decarboxylase encoding gene (*pdC*) in a glucose-suppressed mutant and fermented it for 168 h in a 10 L bioreactor, generating 55.38 g/L of liamocin and 25.10 g/L of cell dry weight from 117.27 g/L of glucose. Olzhausen et al. (2021) increased acetyl-CoA biosynthesis in *Saccharomyces cerevisiae* (*S. cerevisiae*) by overexpressing a dysregulated pantothenic acid kinase gene and engineering the coenzyme A biosynthetic pathway. Wegner et al. (2021) increased coenzyme A biosynthesis by overexpression of pantothenic acid kinase and pantothenic acid supplementation, further increasing production to 3,830 ± 120 mg/L. Yu et al. (2021) enhanced γ-aminobutyric acid production in *E. coli* BL21 by modifying the enzyme of the coenzyme factor pyridoxal 5′-phosphate regeneration pathway.

Satowa et al. (2020) disrupted citrate synthase expression and reduced flux to the tricarboxylic acid cycle, resulting in intracellular acetyl-CoA levels 7-fold higher than in the wild-type strain. This strain produced 8.0 g/L mevalonate from 20 g/L glucose. Zhu et al. (2021) introduced the partial MVA pathway of mevalonate in acetyl-CoA mitochondria and increased mevalonate synthesis in the cytoplasm, which resulted in the squalene titer of 21.1 g/L with a specific squalene titer of 437.1 mg/g dcw. Hayat et al. (2021) introduced heterologous acetylating aldehyde dehydrogenase and phosphoketolase pathways for acetyl-CoA synthesis to enhance acetyl-CoA production to increase the yield of the nerolidol.

By fine-tuning the expression of *pckA* (encoding phosphoenolpyruvate carboxykinase) and redistributing flow between naringenin biosynthesis and cell growth at the isocitrate node, Kim et al. (2022) avoided massive loss of oxaloacetate. When compared to the unoptimized strain, the flux-optimized strain produced 97.02 mg/L naringenin with 21.02 mg naringenin/g acetate, a 27.2-fold increase in naringenin production (and a 38.3-fold increase in acetate production). Su et al. (2022) developed a novel strategy to balance acetyl-CoA metabolism and increase the amount of downstream products. First, the combination of acetaldehyde dehydrogenase and acetyl-CoA thiolase was optimized to redirect acetyl-CoA flux to the target pathway, resulting in a 21-fold increase in mevalonate production. Secondly, growth defects were mitigated by pathway engineering and evolutionary engineering to achieve a 10-fold increase in maximum productivity. ACC was then dynamically down-regulated as a complementary acetyl-CoA pathway, resulting in a more than 2-fold increase in yield. Finally combining the most efficient and complementary acetyl-CoA pathway, the final strain produced 68 mg/g CDW lycopene.

### NAD(P)H/NAD(P)^+^

Many carbon metabolic pathways in microorganisms can synthesize NAD(P)H. Among them, the pentose phosphate pathway is the main metabolic pathway for the synthesis of NADPH, and glycolysis is the main metabolic pathway for the synthesis of NADH. Under aerobic conditions, pyruvate is changed to acetyl-CoA and NADH catalyzed by the pyruvate dehydrogenase complex, and the tricarboxylic acid cycle can generate NADH and NADPH. Non-metabolic coupled expression NADH oxidoreductase may lead to disruption of cellular metabolism, which in turn affects microbial growth and the synthesis of metabolites. Reconstructing the cofactor production pathway can effectively avoid the resulting disruption of cellular metabolism. This section focuses on the metabolic regulatory strategy of NAD(P)H/NAD(P)^+^ and its application in biosynthesis. Some of the NAD(P)H/NAD(P)^+^ regulatory strategies and their applications are listed in Table 2.

**Table 2:** NAD(P)H regulatory strategy.

Cofactors	Chassis cells	Metabolites	Production	References
NADPH	*Corynebacterium glutamicum*	Lysine	Lysine production was significantly improved	Wang J. et al. (2020)
NADPH	*Escherichia coli*	Ethyl S-4-chloro-3-hydroxybutyrate,	A 2.8-fold improvement in reaction yield to the control	Yuan et al. (2022)
NADPH	*Aspergillus niger*	Glucoamylase	The yield of GlaA increased by 65% and 30%, respectively	Sui et al. (2020)
NADPH	*Saccharomyces cerevisiae*	Phenolic acid	Phenolic acid improved by 45%	Chen et al. (2022)
NADPH	*S. cerevisiae*	Bitterness	OA production increased by over 60%	Wernig et al. (2021)
NADH	*Lactobacillus casei*	EPS	Its yield reached 219.4 mg/l, an increase of 46% over the wild-type strain	Li et al. (2015)
NADH	*E. coli*	Xylitol	The highest xylitol productivity of 6.37 g/L/h can be obtained under optimal transformation conditions	Jin et al. (2019)
NADPH	*E. coli*	1,3-Butanediol	The yield is 0.6 mol/mol glucose, which corresponds to 60% of the theoretical yield	Liu et al. (2021a)
NADPH	*E. coli*	ε-Caprolactone	The yield of ε-caprolactone was 0.80 mol/mol	Xiong et al. (2021)
NADH	*E. coli*	L-xylulose	The final concentration and productivity reached 48.45 g/L and 2.42 g/L/h, respectively	Lv et al. (2021)
NADH	*S. cerevisiae*	L-lactic acid	produced 37.94 g/L LA with a yield of 0.66 g/g	Li F. et al. (2022)
NADPH	*E. coli*	Tryptophan	Produced 1.710 g/L of tryptophan, 2.76 times more potent than the parents	Li et al. (2020)
NADH	*A. niger*	Glucoamylase	19% increase in glucoamylase activity	Li L. et al. (2022)
NADPH	*E. coli*	(*S*)-Equol	The conversion of (S)-Equol was about 85.9%	Deng et al. (2022)
NADPH	*Bacillus* sp.	3-PHB	PHB accumulation increased by 2.67-fold	Rami Reddy Tadi et al. (2021)

#### Pentose phosphate pathway

The pentose phosphate pathway(PPP) is the main metabolic pathway for NADPH synthesis, and the key enzymes are glucose-6-phosphate dehydrogenase (G6PDH) and 6-phosphogluconate dehydrogenase (6PGDH), so overexpression of G6PDH and 6PGDH can effectively increase the flux of PPP and intracellular NADPH levels. The transcriptional regulator *Cgl2680* is an important regulator of NADPH levels and L-lysine biosynthesis in *C. glutamicum*. By blocking the glycolytic pathway and overexpressing the PPP in lysine-producing strains and knocking out the gene *Cgl2680*, *Cgl2680*-deficient strains of *C. glutamicum* showed increased production of L-lysine and L-leucine as well as increased H_2_O_2_ tolerance (Wang L. et al., 2020). Insufficient intracellular NAD(P)H is a major issue limiting the reduced product synthesis by whole cell biocatalysis or microbial cell factory. Yuan et al. (2022) improved the PPP by overexpressing additional G6PDH encoding gene *zwf* and glucokinase encoding gene *glk*, thereby increasing the supply of NADPH, and intracellular NADPH content increased significantly from 150.3 to 681.8 mol/L, which was 4.5-fold higher than the control. It was applied to enhance the reduction process of 4-chloroacetoacetate to generate ethyl S-4-chloro-3-hydroxybutyrate, resulting in a 2.8-fold improvement in reaction yield. Sui et al. (2020) enhanced overexpression of the *gndA* gene (G6PDH) and *maeA* gene (NADP-dependent malic enzyme) in the PPP, which increased intracellular NADPH by 45% and 66%, respectively, while glucoamylase yields increased by 65% and 30%, respectively. The regeneration of NADPH was promoted by the non-oxidative step of PPP, which facilitated the synthesis of phenolic acid, and the NADPH regeneration strategy increased phenolic acid by 45% compared to the initial strain (Chen et al., 2022). Wernig et al. (2021) redirected glucose flux to the oxidative branch of the PPP and overexpressed heterologous phosphoketolase/phosphotransacetylase to improve the availability of NADPH and acetyl-CoA in the strain while eliminating eight-carbon fatty acid octanoic acid degradation, and these modifications resulted in more than 60% increase in octanoic acid production during glucose consumption compared to the parental strain.

#### New pathways for cofactor generation

Synthetic biology has provided technical principles for the optimization of key enzymes of metabolic pathways and improvement of cofactor regeneration pathways, and has increased the efficiency of carbon and energy utilization. It was shown that the expression of isozymes that do not produce additional cofactors such as NAD(P)H/NAD(P)^+^ can achieve a balance of cofactors while ensuring normal cell growth compared to endogenous enzymes, the introduction of metabolic synthetic pathways of other strains has become an effective means to address the low yield of target products (Chen R. et al., 2020).

Li et al. (2015) cloned a NADH oxidase gene from *Streptococcus mutans* and overexpressed it in *Lactobacillus casei* (*L. casei*) LC2W under the control of the constitutive promoter P_23_, and the NADH oxidase activity of the recombinant strain LC-nox was 0.854 U/mL, which was nearly 20-fold higher compared to the wild type. The overexpression of NADH oxidase resulted in a 22% reduction in lactate production in the recombinant strain. It was proposed that more carbon sources could be saved and used for extracellular polysaccharide (ESP) biosynthesis with a yield of 219.4 mg/L, a 46% increase over the wild-type strain. Babaei et al. (2019) further increased succinate flux by overexpressing genes in the glyoxylate pathway and the oxidative TCA branch, and expressing phosphoenolpyruvate carboxykinase from succinate-producing *Actinobacillus succinogenes*. Transient adaptation to glucose reduced the lag phase of the strain and increased its tolerance to high glucose concentrations. The resulting strain produced 7.8 ± 0.0 g/L succinate in shake flasks without pH control with a glucose yield of 0.105 g/g. Jin et al. (2019) could completely convert 150 g/L xylose to xylitol by co-expression of xylose reductase and glucose dehydrogenase in *E. coli* and could achieve a xylitol productivity of 21.2 g/L/h by *in vitro* biotransformation. The enzyme activity reached 1,533 U/L after optimization of induction conditions. It was possible to completely convert 200 g/L xylose to xylitol, and the highest xylitol productivity of 6.37 g/L/h was obtained under optimal conversion conditions. Tesfay et al. (2021) introduced NAD^+^ regenerating enzyme (NADH oxidase) from *Streptococcus mutans* ATCC 25175 together with xylitol-4-dehydrogenase from *Pantoea ananatis* into *E. coli*, co-expression resulted in an increase in L-xylulose concentration and productivity from xylitol as well as intracellular NAD^+^ concentration. In a 1 L bioconversion system, the final concentration and productivity of L-xylulose from 50 g/L xylitol reached 48.45 g/L and 2.42 g/L·h, respectively.

In addition, screening and modification of enzymes for application in the synthesis of compounds has become a means of enhancing the target product. Shen et al. (2021) identified 6 genes encoding NADPH-depleting enzymes and 19 ATP-depleting enzymes by CRISPRi screening. The deletion of *yahK*(encoding the NADPH-depleting enzyme), and *fecE*(encoding the ATP-depleting enzyme) increased the yield of 4-hydroxyphenylacetic acid from 6.32 to 7.76 g/L. Liu et al. (2021a) first performed screening for key pathway enzymes, then increased NADPH supply by cofactor engineering, followed by optimization of fermentation conditions, and the engineered *E. coli* strain could efficiently produce (R)-1,3-butanediol at a yield of 0.6 mol/mol glucose, equivalent to 60% of the theoretical yield. Lv et al. (2021) showed that the native *pntAB* encoding pyridine nucleotide transhydrogenase in *E. coli* was selected from five NADPH regeneration genes to supplement redox cofactor NADPH for the conversion of *p*-coumaric acid to caffeic acid during ferulic acid biosynthesis. Slivinskaya et al. (2022) modified the cofactor specificity of *E. coli* GAPDH by amino acid substitutions at positions 34, 188 and 189, several mutant enzymes with dual NAD^+^/NADP^+^ cofactor specificity were obtained and their kinetic parameters were determined. Overexpression of genes encoding mutant GAPDHs with dual cofactor specificity produce d in *E. coli* cells producing L-lysine, L-threonine and L-proline resulted in a significant increase in the accumulation of the corresponding amino acids in the culture medium. Xiong et al. (2021) combined alcohol dehydrogenase (ADH) with cyclohexanone monooxygenase (CHMO) in *E. coli* to obtain a self-sufficient NADPH cofactor regeneration system. In addition, by modifying the ribosome binding site, improved variants with better substrate tolerance and higher catalytic activity for ε-caprolactone were created. The best mutant strain produced 0.80 mol/mol of ε-caprolactone using 60 mM cyclohexanol as a substrate. In four sequential batches, the designed whole-cell biocatalyst produced 126 mM ε-caprolactone with a high molar yield of 0.78 mol/mol.

The introduction of new reactions/pathways, or the reconstruction of metabolic pathways followed by the modified key enzymes has also been shown by many researchers to be an effective means of enhancing the target product. Harth et al. (2020) generated the necessary redox cofactor by combining the reduction reaction of GalA with the oxidation reaction of the sugar alcohol sorbitol (which has a higher reduced state than glucose). Gu et al. (2019) reconstructed the metabolic pathway in GlcNAc-producing strains by introducing the pyruvate iron oxidoreductase PorAB, malate dehydrogenase BmqO, and glyceraldehyde-3-phosphate dehydrogenase GoR to achieve the catalytic processes of pyruvate to acetyl-CoA, malate to oxaloacetate and glyceraldehyde-3-phosphate to 3-phosphoglycerate, respectively, avoiding the excess of NADH and thus achieving intracellular redox homeostasis, ultimately increasing the fermentation yield of GlcNAc by 4.06-fold. The yield of Menaquinone-7 (MK-7) in *Bacillus subtilis* is low, and Ding et al. (2022) made MK-7 production of 39.01 mg/L by overexpressing key rate-limiting enzymes such as 1-deoxyxylulose-5-phosphate synthase, isopentenyl-diphosphate delta-isomerase, 1-deoxyxylulose-5-phosphate reductase. Next, after stoichiometric calculation and optimization of the cofactor regeneration pathway, two NADPH regeneration systems were constructed to enhance the endogenous cofactor regeneration pathway, and heterologous NADH kinase was introduced to increase the availability of NADPH in MK-7 biosynthesis. After three Design-Build-Test-Learn cycles, MK-7 reached a concentration of 53.07 mg/L after flask fermentation. In addition, the artificially constructed cofactor regeneration system resulted in a 9.15% reduction of NADH-dependent by-product lactate in the fermentation broth. This led to a reduction in energy loss and an increase in carbon conversion.

#### Cofactor balance regulation

The balance of cofactor concentrations is an important condition for achieving enzyme catalytic efficiency; simply increasing cofactor concentrations alone does not necessarily improve metabolism, but may instead cause an imbalance in the intracellular environment. When the concentration of NADH is too high, the oxygen consumption of cellular respiration increases, substrate consumption is accelerated, and economic efficiency is reduced. The anabolic metabolism of N-acetylglucosamine leads to an excess of NADH, which is utilized for the synthesis of substances such as the NADH-dependent by-product 2,3-butanediol. Gu et al. (2019) first overexpressed NADH oxidase intracellularly to achieve intracellular redox balance, and when NADH oxidase was expressed using the strong constitutive promoter P43, intracellular redox balance could not be achieved due to overexpression of oxidase, and the fermentation yield of N-acetylglucosamine was only 0.86 g/L. Therefore, further regulation of NADH oxidase using promoter engineering resulted in a final fermentation yield of 12.46 g/L of N-acetylglucosamine. Li F. et al. (2022) regulated the redox genes to consume excessive NADH, and lactate production reached 0.04 g/g to 0.37 g/g in YNB medium. Subsequently, strain PK27 produced 37.94 g/L lactate in YPD medium with a yield of 0.66 g/g. Li et al. (2020) expressed mutated *serA* and *thrA* to increase the precursor supply of serine, resulting in the accumulation of 1.380 g/L tryptophan. Finally, to maintain cofactor balance, the genes *sthA* and *pntAB* encoding transhydrogenases were overexpressed. With sufficient amounts of precursors and balanced cofactors, the engineered strain produced 1.710 g/L tryptophan after 48 h of shake flask fermentation, which was 2.76-fold higher than the yield of the parental strain.

#### Other metabolic regulatory pathways

Insufficient supply of cofactors can limit the production of metabolites, and enhancing the cofactor synthesis pathway can address this limitation well. Li F. et al. (2022) led to a further 19% increase in glucoamylase activity by overexpressing mitochondrial NADH kinase (AN17) and malic enzyme (MaeA). Deng et al. (2022) optimized a cascade system by regulating the intensity of NADPH gene expression to produce a higher (S)-equol titer (3418.5 mg/L) with a conversion rate of approximately 85.9%. van Aalst et al. (2022) functional expression of the gene in anaerobic, slow-growing chemostat cultivation on glucose-sorbitol mixtures resulted in a 12-fold higher co-consumption rate of sorbitol than that observed in sorbitol-consuming reference strains. Rami Reddy Tadi et al. (2021) metabolic engineering of *Bacillus megaterium* through co-expression of the precursor (*phbRBC*) and NADPH cofactor regeneration (*zwf*) genes resulted in a 2.67-fold increase in PHB accumulation.

### ATP/ADP

Currently, there is a better understanding of both material and energy metabolism, and ATP regulation strategies combined with synthetic biology are increasingly being applied in microbial cells to meet the need to enhance target products, etc. (Chen et al., 2017; Cai et al., 2018; Zhang et al., 2019). This section focuses on the metabolic regulatory strategies of ATP/ADP and their applications in biosynthesis.

#### Regulation of oxidative phosphorylation levels

Under aerobic conditions, ATP is synthesized mainly through the oxidative phosphorylation pathway, and therefore the concentration of ATP can be regulated by regulating oxidative phosphorylation (Liu et al., 2006; Hara and Kondo, 2015). Under anaerobic conditions, NADH is oxidized by actions such as acetaldehyde dehydrogenase or lactate dehydrogenase (Zhu and Shimizu, 2004; Zhang et al., 2006), and substrate level phosphorylation is the main source of ATP (Bai et al., 2020), which can be regulated by regulating NADH levels and thus ATP levels (Xu et al., 2017). Dank et al. (2022) discovered that when lactate was consumed under microaerobic conditions, pyruvate, propionate, and acetic acid were produced first, and when lactate was depleted, pyruvate and propionate were oxidized with 6-fold increase in vitamin B12 concentrations compared to anaerobic conditions, demonstrating the potential of propionate and pyruvate as carbon sources for vitamin B12 production. Thus, under microaerobic conditions, a fed-batch reactor with anaerobically precultured lactate-grown cells was fed propionate, resulting in enhanced biomass and vitamin B12 synthesis. Skorokhodova et al. (2021) investigated the anaerobic generation of pyruvate from glucose in recombinant *E. coli* strains with impaired fermentation ability during respiration. Nitrate functions as an external terminal electron acceptor, and enforced ATP hydrolysis causes the recombinant to consume significantly more glucose while maintaining substrate level conversion of the target product. Pyruvate is generated from glucose in yields of 1.77–1.78 mol/mol under anaerobic nitrate respiration and enforced ATP hydrolysis, and the recombinant is almost depleted of substrate with no or minimal by-product formation. Wang D. et al. (2019) knocked out the gene *por1* encoding mitochondrial pore protein in *Candida utilis*, which increased intracellular NADH and ATP concentrations. The co-production of S-adenosylmethionine (SAM) and glutathione in the recombinant strain increased by 34.9% and 25.1%, respectively. Chen and Tan (2018) employed an ATP regulation strategy in yeast to boost SAM production by regulating NADH availability and oxygen delivery, and the SAM titer reached a high of 55 mg/L after 28 h of cultivation. Moreover, respiratory complex I plays a crucial role in regulating the cell growth and secondary metabolism of *Monascus via* by altering intracellular ROS and ATP levels (Cai et al., 2022).

Furthermore, ATP regulation in microorganisms can be performed by introducing related substrates or by overexpressing/knocking out genes encoding related substrates. Ebrahimzadeh et al. (2022) employed a simultaneous pulsed-feeding method of citrate and glutamate to boost poly-γ-glutamate synthesis in *Bacillus licheniformis*, obtaining approximately 88 ± 4 g/L of γ-glutamate under optimal conditions. Kang et al. (2013) added sodium citrate to *Lactobacillus panis PM1* and increased pyruvate production, which in turn led to an increase in acetate and lactate production as well as Zhou et al. (2010) discovered that increased ATP supply improved *Candida glabrata* tolerance in acidic conditions. Xue et al. (2019) improved pullulan production and enhanced the capacity to bind CO and oxygen in *Aureobasidium melanogenum* P16 by overexpression of the optimized *Vitreoscilla* hemoglobin (VHb) gene and the native flavohemoglobin (FHb) gene. During a 10-liter fermentation, the engineered strains were 42.1%, 15.6%, and 40.0% higher than the parental strain in terms of pullulan, yield and productivity, respectively.

#### Metabolic regulation of ATP-related enzymes

ATP synthesis and regeneration are also closely related to ATP synthase and ATP/ADP-dependent enzymes in key pathways. Knockout of ethanol dehydrogenase in *S. cerevisiae* and overexpression of acetaldehyde dehydrogenase increased glycerol production (Cordier et al., 2007). Overexpression of the oxidase gene *aox* in *A. niger* resulted in increased citric acid production (Hou et al., 2018). Integration of the *Arabidopsis*-derived mitochondrial ATP6 gene into *Candida utilis* could increase intracellular glutathione and SAM production by 46.6% and 28.7%, respectively (Xu et al., 2018). Overexpression of phosphoenolpyruvate carboxykinase and triosephosphate isomerase enhances the pyruvate metabolic pathway in shake flask cultures, increasing phloroglucinol concentrations by 44% and 92%, respectively (Liu W. et al., 2020). Heterologous expression of PCK in *Enterobacter aerogenes* and knockout of glucose phosphotransferase increased succinate production (Tajima et al., 2015). The exogenous non-ATP-dependent pathway replaces the native ATP-dependent pathway in *S. cerevisiae*, resulting in a reconfiguration of the metabolic pathway from ethanol to platform compounds such as acetyl-CoA (Kozak et al., 2016). Yamanaka et al. (2010) controlled acidic pH, which stimulated ATP-dependent ε-poly-l-lysine synthase activity and largely enhanced the synthesis of the target compound ε-poly-l-lysine.

## Summary and outlook

NADH, which interacts with related enzymes to affect ATP production under both aerobic and anaerobic conditions, is closely related to ATP synthesis (Hara and Kondo, 2015; Bai et al., 2020). Under aerobic conditions, NADH is oxidized *via* the electron transport chain to produce ATP, with oxygen serving as the terminal electron acceptor, while under anaerobic conditions, NADH is oxidized *via* the fermentation pathway by the action of acetaldehyde dehydrogenase or lactate dehydrogenase, where substrate-level phosphorylation is the main source of ATP. NADH-based strategies to regulate intracellular ATP are easier to manipulate and more efficient, and are suitable for upregulating or downregulating intracellular ATP levels. However, changes in NADH affect the intracellular redox state as well as cell growth metabolism and product synthesis. Therefore, alteration of NADH levels is not a direct method for ATP regulation, and a comprehensive consideration of whether the redox state of the cell is conducive to the synthesis of target metabolites is required.

With the rapid development of cofactor engineering, it has become an important research direction in the field of metabolic engineering and synthetic biology. Currently, the most common cofactors are acetyl-CoA, NAD(P)H/NAD(P)^+^, and ATP, and a variety of corresponding regulatory strategies have been applied to enhance the yield of target products. For example, single or combined metabolic regulatory strategies have been used to regulate cofactors or to specifically modify key enzymes of the cofactor production or consumption pathways. Although the construction of cofactor recycling systems, regulation of cofactor regeneration pathways, and modification of cofactor synthesis pathways have also achieved relatively significant results, these approaches also have various problems and drawbacks, such as the precise dynamic regulation of cofactors, the maintenance of a stable redox state in microbial cells, and the lack of understanding of the complex regulatory mechanisms of essential metabolism, etc., which hinder their application in practical production. In the future, it is expected to continue research on the regulation of cofactors to maximize and rapidly direct the metabolic flow to the target metabolites.

In addition to the research on the regulation of cofactors, some progress has been made in the development of noncanonical cofactor systems in microorganisms. Black et al. (2020) demonstrated the development of an irregular redox cofactor system based on nicotinamide mononucleotide (NMN^+^). The key enzyme in this system is a calculated glucose dehydrogenase, which can be used to support a variety of redox chemistry *in vitro*. This work proves the effective use of noncanonical cofactors in biocatalysis and metabolic pathway design. Recent findings reignite the interest in engineering enzymes using noncanonical cofactors, which exhibit excellent industrial properties *in vitro* and can achieve specific electron transport *in vivo* (King et al., 2020). Wang et al. (2021) reprogrammed the substrate binding pockets of nicotinic acid mononucleotide (NaMN) adenylate transferase to combine to create NCD synthase (NcdS) so that cytidine triphosphate and nicotinamide mononucleotides exceed their regular substrates ATP and NaMN, respectively. Only the overexpression of NcdS in the model host *E. coli* promoted the production of intracellular NCD and reached a higher NCD level as high as 5.0 mM through further regulation. Finally, L-malic acid was converted into D-lactic acid by metabolic circuit mediated NCD connection, which confirmed the self-sufficiency of non-natural cofactors. NcdS and NCD-linked enzymes provide unique tools and opportunities for interesting research in chemical biology and synthetic biology.

## Author contributions

YS and XL conceptualized the review, analyzed the data, and helped to write the manuscript. LJ, TZ, and BL helped to write the manuscript and prepared the figures. All authors contributed to the article and approved the submitted version.

## Funding

This work was supported by the National Key Research and Development Program of China (2021YFC2102700), the National Natural, Science Foundation of China (31922070 and U2106228), the Jiangsu Synergetic Innovation Center for Advanced Bio-Manufacture (XTC2205), the Natural Science Foundation of the Jiangsu Higher Education Institutions of China (22KJB550008), and Scientific Research Start-up Fund for Introduced Talents of Anhui Polytechnic University (2022YOO068).

## Conflict of interest

The authors declare that they have no known competing financial interests or personal relationships that could have appeared to influence the work reported in this paper.

## Publisher’s note

All claims expressed in this article are solely those of the authors and do not necessarily represent those of their affiliated organizations, or those of the publisher, the editors and the reviewers. Any product that may be evaluated in this article, or claim that may be made by its manufacturer, is not guaranteed or endorsed by the publisher.
